# Quality and safety of genetic testing in Australia and New Zealand: a review of the current regulatory framework

**DOI:** 10.1186/1743-8462-3-13

**Published:** 2006-11-08

**Authors:** Imogen L Goold, Amy Pearn, Silvana Bettiol, Angela Ballantyne

**Affiliations:** 1 Intern, Human Genetics Programme, Department of Chronic Diseases and Health Promotion, World Health Organization; St Anne's College, Oxford OX2 6HS, UK; 2 Intern, Human Genetics Programme, Department of Chronic Diseases and Health Promotion, World Health Organization; Molecular Genetics Laboratory, Children's and Women's Health Centre of British Columbia, 4480 Oak St., Vancouver, BC, V6H 3V4, Canada; 3 School of Medicine, Discipline of Pathology, University of Tasmania, 43 Collins Street, Hobart, Tasmania 7000, Australia; 4 Genetics Ethics Officer, Human Genetics Programme, Department of Chronic Diseases and Health Promotion, World Health Organization; Centre For Human Bioethics, Monash University, Clayton, Victoria, 3800, Australia

## Abstract

This paper provides an overview of the regulation of quality assurance for genetic testing in Australia and New Zealand and outlines the steps currently being taken to critically appraise and improve the regulatory framework in each country. It aims to contextualize this framework within the broader context of quality and patient safety concerns; and to draw together the concerns and recommendations of the various organizations that have been working to improve quality assurance in this area.

## Background

Genetic tests have the capacity to generate information that may have a profound effect on the tested individual. Test results may affect the individual's treatment decisions, and choices about reproduction, as well as having potential implications for his or her family members. Given this, it is vital that genetic testing occur within a framework that promotes and protects patient safety and well being. One aspect of this framework is quality assurance, which refers to measures to ensure the quality and consistency of genetic testing.

The purpose of this paper is to provide an accurate, up to date picture of the current state of the regulatory framework governing quality and safety of genetic testing in Australia and New Zealand. We hope that, given the ongoing reviews of and changes to this regulatory framework, this summary will provide a useful snapshot of the current regulatory environment for those working with or interested in genetic testing and patient safety.

## Genetic testing in Australia and New Zealand

Genetic tests are preformed by a range of private and public organisations in Australia and New Zealand. As of 2005, over 220 genetic tests were available in Australia and 45 laboratories were providing genetic testing services [3]. These laboratories are generally attached to or affiliated with a public hospital or university. Tests are also provided by private genetic testing or pathology laboratories. In addition, some government departments have laboratories that conduct genetic testing, for example forensic laboratories. Some tests are also carried out in research laboratories, particularly tests that are still in the research phase or for which there is little demand.

Seven laboratories in New Zealand offer diagnostic testing for approximately 75 genetic disorders [3]. The two main laboratories are the Diagnostic Genetics Department at Auckland City Hospital, and Canterbury Health Laboratories, Christchurch. Outside major city centres, testing services are provided through outreach clinics. The current range of genetic tests available in New Zealand depends on clinical demand, the focus of individual laboratories and the available funds from each District Health Board (DHB).

## What is quality assurance in genetic testing?

Quality assurance in genetic testing refers to measures to ensure that laboratories adhere to high standards of care and undertake testing that is justified and accurate. Quality assurance in genetic testing fits within the broader of concept of patient safety and wellbeing, as it is one element of a range of strategies used within the healthcare system to ensure that patients receive appropriate, effective treatment on a voluntary and informed basis. These strategies include pre-and post-test measures, such as effective identification of individuals at increased risk of a genetic disorder, genetic counselling, appropriate privacy and confidentiality regulation and/or legislation. Genetic tests can reveal highly personal information about an individual and in many cases other family members, hence appropriate measures should be in place to protect the privacy and confidentiality of the results, to prevent unauthorised access and possible misuse of this sensitive information. This paper focuses on the regulation of quality assurance of genetic testing, so only the test stage of the testing process is examined in detail.

At the test stage, measures to ensure quality assurance fall into two categories – (1) the safety, accuracy and utility of a test; and (2) the competency of the laboratory and its staff in performing that test. The safety, accuracy and utility of a test are important issues in patient safety because a lack of any one of these characteristics may lead to the patient receiving misleading or unhelpful information. For example, there is debate about whether genetic tests should be preformed if there is not available treatment for the condition. The genetic condition of Huntington's disease provides a relevant example. Although there is currently no cure for Huntington's disease, some at risk people choose to have the genetic test and use the information to inform his or her reproductive decisions. Tests are judged in relation to analytical validity, clinical validity, and clinical utility to ensure they are safe and accurate. Analytic validity is the ability of a test to detect the trait it seeks to measure. Clinical validity is the capacity of the test to predict a specific clinical outcome. Clinical utility refers to the actual usefulness of the test in improving health and well-being of the persons tested and largely rests on whether the information provided by the test can be followed by effective and safe preventive or therapeutic interventions.

The second aspect of quality assurance at the test stage is laboratory competency. If tests are not performed correctly, using the right equipment, or the laboratory staff lack the skills to perform the tests, then the results may be inaccurate. Further, the laboratory should be competent in managing samples and results, maintaining confidentiality and privacy, and in delivering results appropriately. Administrative failures may lead to the loss of results, mix ups or breaches of privacy and confidentiality, harmful to the patient's interests. It is therefore important to ensure that laboratories are competent to undertake and manage the tests they offer.

## Quality assurance regulation general framework

Genetic testing in Australia and New Zealand is subject to the more general scheme of laboratory and test accreditation, although some specific regulations and guidelines do apply. This reliance on a generic regulatory framework is largely due to the fact that the aspects of genetic testing and genetic information that differ from general testing and medical information are most relevant at the pre- and post-test stage. For example, genetic counselling is necessary prior to, and following, some genetic tests due to the particular ramifications of the results, but this is regulated largely through guidelines for professionals working in genetics, rather than at the test stage [see, eg, [4]].

This general quality assurance scheme is comprised of legislative requirements, accreditation standards, and guidelines developed and administered by a number of organizations. In both Australia and New Zealand, the national government acts as the primary regulatory body for healthcare services through a national health department in collaboration with a number of government advisory bodies. Regulatory bodies also include laboratory and professional accreditation bodies, and consumer protection agencies. In addition, professional organizations act as advisory bodies, offering independent guidelines and standards of practice. There is some overlap between the Australian and New Zealand schemes.

### General healthcare framework for Australia

The regulatory framework governing the provision of genetic tests sits within the broader healthcare system and so it is important to have a sense of the way health services are provided and funded in Australia and New Zealand. The Australian health care system is largely regulated (and partially funded) at the State and Territory level; however the Federal Government also provides funding and has a regulatory role. Medical services are provided through both the public and private sectors, and public subsidies (Medicare benefits) are available for many services.

**Figure 1:**
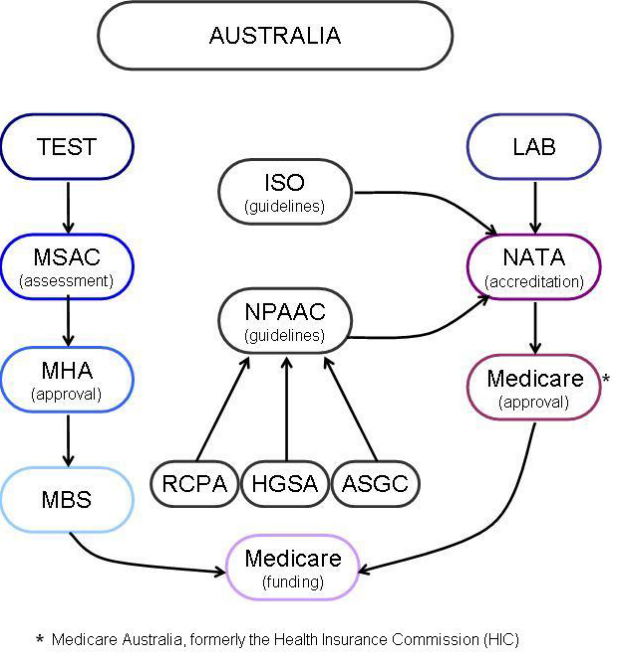
Regulation and accreditation pathways for genetic testing in Australia.

The Federal government regulates the provision of pathology services only indirectly through the administration of the Medical Benefits Schedule (MBS) by withholding subsidies for pathology services from laboratories that are not appropriately accredited [[5], p4]. It also regulates specific *tests*, whereby only accredited tests are subsidised. The States and Territories do have the power to regulate pathology services, however only Victoria has enacted specific legislation in this area [6]. The test and laboratory accreditation schemes are outlined below; however Figure 1: Regulation and accreditation pathways for genetic testing in Australia provides a summary of these schemes and their interaction.

### General healthcare framework for New Zealand

The Ministry of Health Manatû Hauora (MOH) is the New Zealand governmental agency that issues guidelines and policy for medical services throughout New Zealand [7]. The MOH distributes funding to District Health Boards [8] which are in turn responsible for funding and delivering health and disability services in their district. DHB laboratories perform the majority of genetic tests, and are usually based in public hospitals.

**Figure 2:**
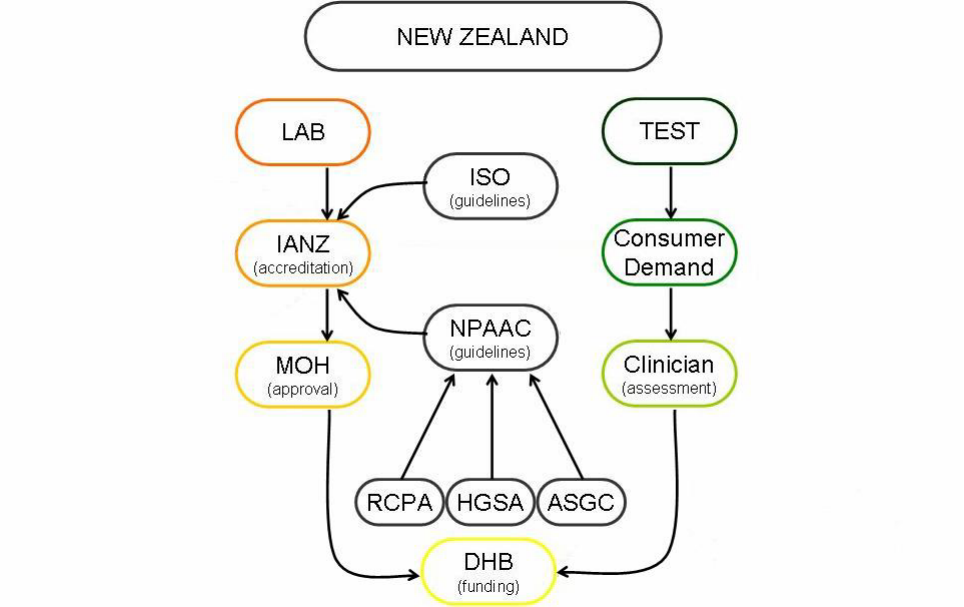
Regulation and accreditation pathways for genetic testing in New Zealand.

Testing services and laboratories are regulated to some degree by the MOH as part of its responsibility for maintaining the National Health Service. The MOH regulates some requirements for laboratory quality and auditing, as well as defining the mechanisms through which laboratories are funded [[9], p9]. Laboratory accreditation in New Zealand is administered by an agency called International Accreditation New Zealand (IANZ). The New Zealand schemes for introducing new tests and ensuring laboratory quality standards are discussed below, however the regulation and accreditation pathways are also summarised in Figure 2: Regulation and accreditation pathways for genetic testing in New Zealand.

## Test accreditation

### Australia

In Australia, the use of tests is regulated partially through funding incentives provided by the Federal Government. For tests to attract a government subsidy, an application must be made to the Medical Services Advisory Committee (MSAC) [10], which advises the Federal Government on new medical technologies and procedures. MSAC bases its assessment on the safety, effectiveness (including validity and utility), and cost-effectiveness of the test in accordance with the MSAC guidelines [11]. MSAC makes recommendations to the Federal Minister for Health and Ageing, who then decides whether the test should receive public funding. If successful, the test will be listed on the MBS and Medicare benefits will be available.

This system creates an incentive for laboratories to provide only tests that have been accredited, as otherwise patients will be unable to apply for Medicare benefits to offset the cost of the test. It does not, however, prevent unaccredited tests from being offered and therefore does not wholly protect patient safety.

Further, the MSAC submission process can be both time-consuming and expensive, which may discourage the submission of some tests where the process may not be cost-effective. Completing the MSAC application process can be labour intensive and time consuming as applicants must attend a pre-lodgement meeting and then complete a forty-three page application form. In completing the application, the applicant should make reference to the 2005 guidelines which are 100 pages long. This may discourage manufacturers of diagnostic tests for rare genetic conditions and a small potential market from applying to MSAC for Federal funding [40].

### New Zealand

Unlike Australia, New Zealand has not introduced a formal system for validating new genetic tests and the MOH has no formal role in assessing new tests. Rather, new tests are introduced in response to clinical demand, or as a result of the individual interests of each laboratory [[9], p8]. Each DHB is responsible for funding genetic tests, and are therefore responsible for the quality of testing in their regions. Genetic tests are also not listed on the Laboratory Services Schedule, which lists tests available for public funding in New Zealand, as determined by the Laboratory Services Advisory Group. There is also no advisory body in New Zealand responsible for determining whether a genetic test should be publicly funded [[38], p13].

Despite this lack of formal assessment of new tests, those that are offered in New Zealand tend to be for common genetic conditions and are usually well validated. Where a patient requires a test for a less common condition, the sample will often be sent to a laboratory outside New Zealand [[9], p9]. Further quality assurance is provided through the laboratory accreditation scheme, as a laboratory cannot receive healthcare subsidies for any test unless it has received IANZ accreditation to perform that test [[9], p13].

### In vitro diagnostic devices

It should be briefly noted that a new scheme for regulating in vitro diagnostic devices (IVDDs), which will cover some genetic tests, is being developed by the Australian Therapeutic Goods Administration (TGA). Development of the scheme was initiated in January 2002 by the TGA, and an agreement in principle to the proposed regulatory framework for IVDDs was made by Australian Health Ministers' Advisory Council (AHMAC) members on 23 October 2003. An outline of the framework can be found at the TGA website [47].

The TGA is currently developing risk-based classifications for IVDDs, and this scheme will include genetic tests. An IVDD will be placed in one of four risk classes:

• Class I: no public health risk/low personal risk;

• Class II: low public health risk/moderate personal risk;

• Class III: high personal risk/moderate public health risk;

• Class IV: high public health risk.

The TGA has given examples of genetic tests that will fall into some of these classes. Class II IVDDs are tests "that detect the presence or exposure to infectious agents that are not easily propagated in the Australian population or that cause self-limiting diseases" and present a moderate individual risk, where the test is not intended to be the sole diagnostic method or "where an erroneous result rarely puts the individual in immediate danger". This will include some genetic tests such as thrombophilia mutation screening tests.

Class III IVDDs are tests that provide the "critical, or sole, determinant for the correct diagnosis", where "an erroneous result would put the patient in an imminent life-threatening situation, or would have a major negative impact on outcome". The TGA also states that these are tests that "may also present a high individual risk because of the stress and anxiety resulting from the information and the nature of the possible follow-up measures". This class of IVDDs will include predictive genetic screening tests for conditions that will usually have "a substantial impact on the life of the individual", such as tests for phenylketonuria (Guthrie test), Huntington's Disease, Cystic Fibrosis [47].

### Some comparisons

The major difference between the Australian and New Zealand systems is that Australia has developed a centralised process for assessing new genetic tests before they can be offered to patients. Such a system is useful, as it ensures that all tests are consistently evaluated for analytical and clinical validity as well as clinical utility before they are provided to the public. However, it appears that in New Zealand, for the most part, only tests that have been well validated either within the country or elsewhere are offered, and at present there is no evidence that this lack of formal regulation is highly problematic. This may be because health services in New Zealand are more locally integrated than their Australian equivalents.

## Laboratory accreditation

### Australia

The Federal Government uses funding incentives to encourage laboratories to become accredited (and hence maintain adequate laboratory standards). To receive Medicare payments for medical services, a laboratory must be accredited in the relevant testing services, comply with the NPAAC guidelines and be designated an Accredited Pathology Laboratory.

Accreditation of testing services is administered by the National Association of Testing Authorities, Australia (NATA). NATA is an independent, private, not-for-profit company operating as an association. It is owned and governed by its members (registered laboratories) and representatives from industry, government and professional bodies (such as the Royal College of Pathologists). NATA, as a member of the Asia-Pacific Laboratory Accreditation Cooperation (APLAC), regularly participates in audits by, and of, its mutual recognition partners in Europe, North America and the Asia-Pacific region. NATA operates an accreditation scheme for laboratories and is the Federal Government-endorsed accreditation body for establishing competent laboratory practice. This was outlined in the Memorandum of Understanding between NATA and the Commonwealth government [13].

Laboratories apply directly to NATA for accreditation in types of medical testing. NATA's medical testing accreditation scheme is based on the relevant guidelines issued by the National Pathology Accreditation Advisory Council (NPAAC). NPAAC consists of representatives from the DHA, the State and Territory departments of health, and from peak professional organizations including the Royal College of Pathologists of Australasia (RCPA) and the Human Genetics Society of Australasia (HGSA), among others. NPAAC advises the Commonwealth, State and Territory Health Ministers on matters relating to the accreditation of pathology laboratories.

NATA accreditation standards are developed with reference to international standards (such as ISO) for laboratory competency in consultation with the RCPA. Such international standards are incorporated by a requirement that NPAAC guidelines be read "with" the standards, such as the *Laboratory Accreditation Standards and Guidelines for Nucleic Acid Detection Techniques *which are to be read with AS ISO/IEC 17025:1999, *General requirements for the competence of testing and calibration laboratories*. This adds an extra layer of quality assurance to the requirements put in place by NATA and NPAAC.

Once laboratories apply for accreditation, NATA conducts an inspection to determine whether they satisfy NPAAC requirements and, if successful, they then become members of NATA. They must undergo periodic future inspections to maintain accreditation [[5], p2]. In accrediting a laboratory, NATA endorses its competency to undertake testing, which can include genetic testing. Professionals from the pathology industry volunteer to act as peer reviewers for NATA accreditation assessments. Peer reviewers undergo accreditation assessment training and work with a NATA staff officer to ensure objectivity and consistency [[5], p11].

To obtain NATA accreditation and receive Medicare benefits laboratories must comply with the standards and guidelines released by NPAAC [14]. NPAAC standards and guidelines cover many aspects of laboratory practice including: laboratory ethics; quality systems; staffing, supervision and consultation; facilities; test ordering, analysis, and follow-up; occupational health and safety; and internal and external auditing for quality assurance [15]. In particular, NPAAC's *Standards for Pathology Laboratories *requires that patient wellbeing and confidentiality be the primary considerations of the laboratory when performing tests [[15], Standard 1]. This means that no person should disclose patient or test information to anyone other than the requesting medical practitioner, or other medical practitioner currently treating the patient, except in some defined circumstances outlined by state and federal privacy legislation. NPAAC requires that human samples, tissues and remains be treated with due respect. Finally, it is the responsibility of the laboratory to ensure that the quality of their work is not affected by any improper pressure, be it financial, commercial, or otherwise.

NPAAC standards require laboratories to undergo external quality assurance testing programs, such as those provided by the RCPA, and to perform to an acceptable standard [[15], Standard 9]. Data from these external programs are made available to NATA during assessment [[5], p14]. Where there is no external proficiency program available, laboratories are required to undergo inter-laboratory comparisons and/or analysis of reference and control materials [[15], Standard 9].

Once accredited, to receive Medicare benefits for tests, a laboratory then must apply to the Minister for Health and Ageing (the Minister) via Medicare Australia for approval to become an Accredited Pathology Laboratory (APL), as only APLs may obtain Medicare benefits for pathology services provided [[12], s 16A(2)(b)]. The approval process and requirements are is provided by the *Health Insurance Act 1973 *(Cth) (HI Act) [[12], s 23DN(1), (2)]. Accreditation decisions must be made in accordance with the HI Act and the *Health Insurance (Accredited Pathology Laboratories-Approval) Principles 1999 *(Cth). Laboratories applying for accreditation must have undergone a NATA inspection prior to application and include the NATA inspection report with their application. Only those pathology services approved by NATA will be eligible for Medicare benefits if accreditation is obtained.

Without NATA accreditation, and subsequent approval of the laboratory and personnel by Medicare Australia, laboratories are unable to access the benefits of Medicare payments for their services. In the case of a breach of the principles outlined by NPAAC, NATA, and Medicare Australia, Medicare benefits and accreditation may be removed. Inability to provide tests at subsidised rates may adversely affect a laboratory's capacity to provide competitively-priced tests, and hence the revocation of Medicare benefits is a strong incentive for laboratories to achieve accreditation.

Until recently, Victoria had an independent structure for accreditation of all pathology laboratories in the state, regardless of whether they seek Medicare payments [16]. The Pathology Services Accreditation Board, on behalf of the State Minister for Health, governed accreditation procedures and legislation as defined by the *Pathology Services Accreditation Act 1984 *(Cth) in accordance with NPAAC standards and the NATA/RCPA scheme [[17], s9]. However, this act was repealed in 2003 by section 14(1) of the *Health Legislation Amendment Act 2003 *(Vic), and the Board consequently dissolved in January 2004 [43].

#### Genetics-specific regulation and accreditation

NPAAC standards do make some specific provisions for the unique challenges raised by genetic testing. Previously, under the *Laboratory Accreditation Standards and Guidelines for Nucleic Acid Detection Techniques *2000 version, NPAAC recognised that "the implications of laboratory diagnosis of genetic disease are different from those of many other areas of laboratory testing" and as a result draws a distinction between two types of testing – diagnostic genetic tests (Class A) and predictive, carrier and prenatal genetic tests (Class B). For Class A tests, only verbal consent is required and there is no requirement for pre-test counselling. By contrast, Class B tests are carried out on non-symptomatic patients who must receive pre- and post-test counselling and must provide formal, written consent.

In addition, laboratories were required to take responsibility for ensuring the formal consent has been obtained and counselling provided. For Class B tests, where the laboratory suspects that proper consent has not been obtained, it is required to contact the referring practitioner to ensure informed consent has been obtained before it may undertake testing [[20], para 1.2]. Under the same standard, laboratories are prohibited from providing patient-initiated tests, such as mail-order testing.

This description of DNA testing for inherited genetic disorders – divided into Class A and Class B – has been revised in version 6.1 of the NPAAC standards and guidelines implemented in August 2006 [1,42]. This change was deemed necessary because, a particular test could move between Class A and Class B on a case by case basis, depending on its use and circumstances, which has caused confusion. The latest draft of the NPAAC guidelines for Nucleic Acid Detection (set to be approved in 2006) has attempted to dispel this confusion by changing the classification system. The Level 1 (standard DNA test) and Level 2 DNA test (complex issues) categorisation allows classification to vary regardless of the test based on the implications of the testing situation.

Level 1 DNA tests (standard) include a) DNA testing for confirmation of diagnosis where the patient has a clinical diagnosis, symptoms, or a family history of an established inherited disorder or any other DNA test that doesn't fall into level 2; b) Neonatal screening programs.

Level 2 DNA tests are considered those tests which could potentially to lead to complex clinical issues. Level 2 tests would include predictive or presymptomatic DNA testing, and tests for conditions for which there is no simple treatment. In these cases, specialised knowledge is often necessary to determine the need for testing (i.e. the test should be requested by a specialist rather than any physician). Level 2 tests should be accompanied by both pre- and post-test professional genetic counselling and may also require specific written consent [[42], Table 1.1].

Version 6.1 of the NPAAC document will require laboratories to review the categorisation of DNA tests for human inherited disorders with the guidance of representative professional bodies. Classification of a test as Level 1 or Level 2 will take into consideration "resources, current knowledge, circumstances, the type of condition being tested for, and the implications of the DNA test result for the patient and family".

Laboratories providing genetic testing in Australia are also covered by the influential, if not binding, information papers, guidelines and principles released by the National Health and Medical Research Council which outline some of the ethical aspects of genetic testing [see, eg, [21]], and policies released by the HGSA [22].

### New Zealand

International Accreditation New Zealand (IANZ) is the Crown-owned, user-funded authority providing accreditation for a range of laboratories and related technical services in New Zealand, including pathology laboratories [9]. IANZ Laboratory Accreditation for Medical Testing complies with *NZS/ISO 15189:2003 Medical Laboratories – Particular Requirements for Quality and Competence *[23]. This standard replaced the New Zealand Code of Laboratory Management Practice for all IANZ accreditation as of 1 January 2004 [24]. The IANZ accreditation system and the specialist technical peer-review process include the assessment of laboratory staff. IANZ further requires that laboratories offering genetic testing comply with the Australasian NPAAC guidelines [see list of applicable guidelines at [25]].

In addition, both the HGSA and the RCPA offer quality assurance assessment for genetic tests by carrying out proficiency testing for some common tests [[9], p13]. Many laboratories also participate in College of American Pathologists (CAP), and/or European Molecular Quality Network (EMQN) programs [Personal communication (March 14, 2005) Dr. Karen Snow Bailey, Director of Diagnostic Genetics, LabPlus, Auckland District Health Board, New Zealand]. IANZ has entered into a mutual recognition arrangement with NATA (the Australian accreditation body) recognizing equivalency of their standards of accreditation [26]. New Zealand laboratories are therefore also subject to NPAAC guidelines, as compliance with these is a prerequisite of NATA accreditation.

The IANZ reports annually to the MOH. If the ministry is informed of a breach of the IANZ guidelines, action is taken through the funding agreements with the DHBs, rather than by taking action against the individual laboratory. The DHB is responsible for ensuring the quality of the laboratories to which it provides funding [9].

#### Genetics-specific regulation and accreditation

As IANZ uses NPAAC standards, New Zealand laboratories are subject to the same standards in relation to genetic testing as described below. New Zealand laboratories are also subject to HGSA policies, but not to NHMRC guidelines.

### Australia and New Zealand overlap and the joint Trans Tasman scheme

**Figure 3:**
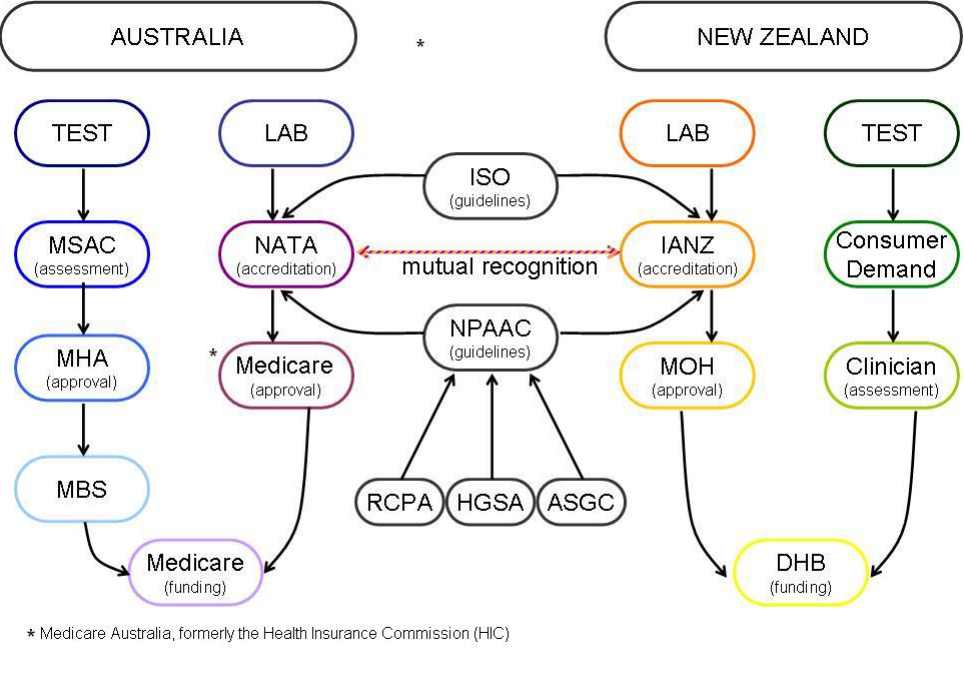
Combined regulation and accreditation pathways for genetic testing in Australia and New Zealand.

From the preceding discussion, it becomes clear that Australia and New Zealand have integrated their two quality assurance systems to some degree. This integration is summarised in Figure 3: Integration of regulation and accreditation pathways for genetic testing in Australia and New Zealand.

Recently, the two countries took steps towards further integration of their schemes in December 2003 by accepting a joint proposal for a Trans-Tasman agency for the regulation of therapeutic products [[27]; 28]. The proposed agency will replace Australia's TGA and New Zealand's Medsafe, and will include regulation of materials for genetic testing and IVDDs. At the time of writing (August 2006), considerable progress towards establishing the agency, to be known as the Australia and New Zealand Therapeutic Products Agency, had been made. The first round of public consultations were held in June 2006 in Australia and New Zealand and the second round is proposed for September 2006 and a third and final round for March 2007 [46]. According to the Therapeutic Products Interim Ministerial Council, the scheme is expected to commence in the second half of 2007 [49].

Establishing the Trans-Tasman agency is an encouraging step. It will improve consistency in practices and streamline quality assurance procedures, which will be particularly important in this region. Samples are often sent between Australia and New Zealand for testing, for example in cases where one country lacks capacity in a particular test, and improved consistency and integration can only further develop quality assurance mechanisms by removing gaps in regulatory coverage. Further, this joint approach will better equip each country to address regulatory issues raised by new, innovative technologies as they emerge [45].

## Reviews of regulatory and accreditation systems

Australia and New Zealand have been pro-active in their attempts to identify and address the problems in their systems of quality assurance with respect to genetic tests. Genetic testing is an area of medical technology that continues to develop rapidly. For this reason, the Australian and New Zealand accreditation schemes are subject to regular reviews to assess whether they effectively ensure appropriate quality standards in genetic testing. These reviews have highlighted a number of areas for improvement. We now describe the findings of recent reviews and present a summary of the common themes of these evaluations.

Note that some of these reviews referred to the Health Insurance Commission (HIC), which from October 2005 has been known as Medicare Australia. These references have been left as they were in the original reviews, however all references to the HIC in this section should be taken as now referring to Medicare Australia. The HIC *Annual Report 04–05 *provides information on the changes to the Commission and the relevant legislative amendments [[44], Ch 2.2].

### Australian reviews

#### Australian Law Reform Commission inquiry

In 2001, the Australian Law Reform Commission (ALRC), a permanent, independent federal statutory corporation providing advice to the Australian Federal Government on areas of law reform, began a comprehensive two-year inquiry into the protection of genetic information in Australia (ALRC Inquiry) [30].

The ALRC Inquiry included a review of some aspects of pathology service provision, and identified a number of concerns in relation to quality assurance and genetic testing, including:

• the lack of independent assessment of non-accredited laboratories;

• failure of current accreditation standards to address issues such as informed consent, privacy, and chain of custody of samples;

• the possible provision of genetic tests direct to the public, without proper consent or counselling [[30], Ch 11].

The ALRC made a range of recommendations directed at addressing these concerns. It recommended that:

• An oversight body for issues around genetic testing and genetic information, known as the Human Genetics Commission of Australia (HGCA) be established [[30], Recs 5–1 to 5–9];

• The HGCA should develop codes of practice and advice relating to the provision of technical and ethical standards for genetic testing services provided direct to the public [[30], Recs 11–2 to 11–7];

• The HGCA should develop genetic testing and counselling practice guidelines, in consultation with HGSA, state genetic services, and other interested parties [[30], Rec 23–2];

• NPAAC should further develop ethical standards for medical genetic testing, in consultation with the NHMRC and the proposed HGCA;

• NPAAC should examine how to assess compliance with accreditation standards in relation to consent, counselling and other ethical considerations; and NATA should develop training programs to equip its officers and peer assessors to verify compliance [[30], Recs 11–2 to 11–4];

• The Commonwealth Government should amend the *Therapeutic Goods Act 1989 *(Cth) to better regulate genetic tests provided directly to the public [[30], Recs 11–2 to 11–5].

To date, only some of the ALRC's recommendations have been implemented. However, one notable success is the Federal Government's commitment in the 2005 Federal Budget to provide $7.6 million over four years to establish an independent expert advisory body on human genetics. The new body, named the Human Genetics Advisory Committee, is now a central committee of the NHMRC [31]. This body satisfies the inquiry's core recommendation for the establishment of the HGCA, and is a significant step towards more effective regulation of genetic testing, including quality assurance. More information about the functions and membership of the committee can be found on the NHMRC website [52]. The Federal Government is currently preparing a response in relation to the remaining recommendations in the ALRC Inquiry report [31].

#### Department of Health and Ageing reviews

Also in 2001, the Federal Department of Health and Ageing (DHA) commissioned a major independent review of the Australian pathology laboratory accreditation arrangements (Pathology Accreditation Review). This review was the first comprehensive evaluation of the arrangements since their introduction [[5], p1]. The review found that "the current Australian pathology accreditation arrangements are fundamentally sound and should be maintained" [[5], Rec 2.1]. However, the review also identified a number of areas in which accreditation could be improved. These included:

• Lack of Federal power to directly regulate pathology services [[5], p4].

• Delays in current management of non-compliance in a small number of laboratories, which have resulted from delays in arranging or conducting NATA/RCPA assessments; delays in referral of non-compliant laboratories to the HIC; due to NATA's internal appeal process; or due to administrative appeal procedures [[5], ppi, 4].

• As most laboratories are regulated through administration of the MBS, the small number of laboratories that do not seek Medicare benefits are unregulated [[5], pi]. As a result, it is difficult for the HIC to enforce compliance with NPAAC standards within these laboratories [[5], p4].

The Pathology Accreditation Review resulted in 37 recommendations, the great majority of which were accepted by the DHA in its response [32]. Many of these recommendations were detailed and focused on very specific aspects of the accreditation process, therefore only a few more general, overarching recommendations are outlined here. In particular, the Pathology Accreditation Review recommended that an evaluation be made of the costs and benefits of enacting State and Territory legislation to complement the national regulatory system. As the States and Territories have the power to directly regulate pathology services, such legislation could address the lack of regulation of laboratories that fall outside the current accreditation system because they do not access MBS subsidies [[5], Rec 2.2]. The ALRC Inquiry made a similar recommendation [[30], Rec 11–1].

Finally, the Pathology Accreditation Review recommended increasing the sanctions open to the HIC to deal with non-compliant laboratories. The HIC should be able to:

• require that laboratories participate in re-inspection;

• to notify the public of laboratories rated 'Non-Compliant with Moderate or Serious Risk';

• to require laboratories to notify referring medical practitioners and/or consumers of their non-compliant status; and

• to revoke Medicare benefits [[5], Rec. 6.1].

In relation to the need to identify an organisation or individual with clear responsibility for the oversight of the pathology quality assurance system, the Australian Council for Safety and Quality in Health Care (ACSQHC) has undertaken preliminary work to consider structures that could oversee standard setting and quality monitoring in this area. Therefore, the Pathology Accreditation Review recommended that the DHA allocate responsibility for the oversight of pathology quality assurance systems to a DHA senior officer until the ACSQHC takes further action [[5], Rec. 6.5].

As a result of this review, the Commonwealth Government has announced moves to improve the standards of pathology laboratory testing and to identify laboratories that do not meet required standards [33]. The DHA confirmed in 2003 that the Australian Health Ministers' Advisory Council Group on Human Gene Patents and Genetic Testing was considering the establishment and implementation of an improved quality assurance and accreditation scheme [[30], p339].

The DHA has instigated two other reviews of the regulation of pathology services. One, the *Review of the Commonwealth Legislation for Pathology Arrangements under Medicare*, was completed in 2002 (DHA Review) [34]; the other, the *Review of enforcement and offence provisions of the Health Insurance Act 1973 as they relate to the provision of pathology services under Medicare *(Enforcement Provisions Review) was completed in August 2005 [35]. These reviews examined in detail some aspects of the regulatory framework for pathology services, including enforcement and offence provisions, compliance arrangements, funding and accreditation.

The DHA Review noted two main challenges relevant to quality assurance in genetic testing, to which the Federal Government has responded positively. First, the government is taking steps review the current qualification requirements and the approval process for Approved Pathology Practitioners to ensure, among other things, regular checks on staff competency (in line with Recommendation 11) [[34], Rec. 11] [36].

Second, the new Pathology Laboratory Principles that came into effect on 1 January 2003 act on part of Recommendation 13 to:

• strengthen links between the NATA accreditation process and approval as an APL;

• strengthen the HIC's powers in relation to laboratories operating below standard; and

• develop links between participation in a quality assurance program and notification of the results [[34], Rec. 13, 36].

The DHA Review also considered there was a need for greater enforcement provisions, recommending the establishment of new offences, strengthening of the Medicare Participation Review Committee and "introducing a system of direct administrative action" by HIC [[34], Rec. 14].

In August 2005, Phillip Fox Lawyers released the government commissioned Enforcement Provisions Review report [50]. This Review was commissioned by the DHA in response to recommendations 14 and 15 of the previous DHA Review. The Enforcement Provisions Review makes a series of recommendations aimed at clarifying and strengthening the provisions of the *Health Insurance Act 1973 *(Cth) (HI Act). While there is not space here to summarise the wide range of issues addressed in the report, its primary recommendations focus on making it clearer that certain relationships between pathology providers and requesting practitioners are prohibited, and ensuring that appropriate enforcement action is available where breaches occur. In particular, the Review clarifies that provision of, or demand for, financial or other incentives for referral for pathology services breaches the letter and spirit of the HI Act, particularly s129AA and s129AAA (p2 Section 1.11). To give one example, the Review recommends that a Medicare benefit be reinstated to cover the costs incurred by general practitioners in collection of samples for pathology testing (particularly in rural and remote areas) (Recommendation 33), which would prevent the provision of incentives by the pathology provider in the form of disposables for sample collection.

In May 2006, the government accepted the bulk of Phillip Fox's recommendations and has and agreed to act on these as swiftly as possible to expedite clarification and strengthening of the enforcement and offence provisions, and simplification of the sanctions process [51]. This will include the recommended amendments to the HI Act, and additions to and consolidation of s129AA and s129AAA (which will likely involve the repeal and replacement of the provisions, rather than amendment). In some cases the government agreed to take action supporting the intent of the recommendation, rather than the specifics proposed by the review. These included Recommendation 2, that the HI Act be amended to enable the HIC to address "serious and imminent risk to public health" by suspending an APL approval without a hearing to the APA. The government agreed, in conjunction with Medicare, to explore options and determine minimum requirements for this action to be taken [51].

#### National Public Health Partnership report

Until July 2006, the National Public Health Partnership (NPHP) was a sub-committee of the Australian Health Ministers' Advisory Council (AHMAC), established under a Memorandum of Agreement between the Federal, State and Territory governments in 2003 [37,53]. During its operation, the NPHP compiled *An Overview of Public Health Surveillance of Genetic Disorders and Mapping of Current Genetic Screening Services in Australia*, released in October 2002, to summarize the current organization and availability of genetic services, and the legislation in place to regulate these services (NPHP Overview).

The report identified a number of issues, and recommended that a national approach be taken to deal with them. The issues it identified that relate to quality assurance in genetic testing were that:

• Existing legislation used to regulate genetic testing is not always specific to genetics. Some states rely on general federal regulations that may be used to cover genetic testing.

• Although there is widespread voluntary compliance with NPAAC, HGSA, and NATA guidelines, there are no direct measures to combat non-compliance.

• The division of healthcare funding between the Federal Government and State and Territory governments raises issues about ensuring equitable access to tests.

### New Zealand reviews

Two major reviews of genetic testing practices have been undertaken in New Zealand in the past three years. The first, a report by Dr Diana Sarfati for the National Health Committee (NHC) [9] released in 2002 (Sarfati Report), provides an overview of genetic testing in New Zealand in relation to international standards of best practice. The report did not focus on identifying areas for improvement nor did it make any recommendations, but did note a number of areas of concern, such as:

• The lack of formal quality control programmes to ensure standards of quality for genetic testing in some laboratories.

• The capacity of current accreditation mechanisms to detect poor laboratory practice.

• The exclusion of research laboratories providing clinical testing from IANZ accreditation requirements, as they may lack quality assurance processes.

• IANZ's lack of power to enforce compliance. For example, there is no requirement for it to contact the MOH when it suspects that a laboratory is not meeting its standards Even when the MOH is informed, its only means of pressing for compliance is funding agreements with DHBs, which are responsible for quality of testing services [9,13,14].

The report also noted that the NHC was working on a framework for assessing new technologies, including genetic tests, that would, among other things, examine their safety and effectiveness [[9], p9].

In 2003, the National Advisory Committee on Health and Disability of New Zealand issued a report for the NHC, *Molecular Genetic Testing in New Zealand *(NHC Report) [38]. This document built on a 1995 report to the NHC on priorities for genetic services in New Zealand [39], and highlighted the areas requiring further development to meet the growing demand for genetic services and to ensure the equitable, safe and appropriate provision of medical genetics services to the New Zealand public. The report noted the lack of a single agency taking an overarching view of genetic technologies and service delivery, an absence that has implications for the effective regulation of quality assurance in relation to genetic testing [[38], p4].

Echoing some of the findings in the Sarfati report, the NHC report highlighted the lack of a coordinated mechanism for developing and evaluating new tests, the exclusion of genetic tests from the Laboratory Services Schedule and the absence of an organisation responsible for making public funding decisions about these tests. It commented on the risks raised by the increasing availability of tests where utility and validity were uncertain or had been inadequately assessed, and suggested these risks were associated with the death of measures to ensure the quality of new tests

[[38], p13].

Problems with quality assurance mechanisms for laboratories were also identified. It found that laboratories carrying out low-volume tests might not be performing tests often enough to maintain competency. Further, as research laboratories need not be IANZ accredited, yet sometimes perform clinical testing services, they may not be subject to adequate quality assurance programmes. The NHC considered that genetic testing particularly challenged the current system of quality assurance, due to its rapid rate of development which demanded more frequent reviews of laboratory competency than presently in place [[38], p18].

Finally, the NHC Report noted that New Zealand needed to consider the particular cultural concerns of the Maori community in developing more effective quality assurance measures. Genetic testing raises two potential concerns for Maori. First, some Maori believe that genetic technology may change the *whakapapa*, a spiritual value that relates to Maori identity and which refers to genealogy, tribal histories and genetic inheritance. Second, that for some Maori it may be culturally inappropriate to send genetic samples outside New Zealand for testing [[38], p23].

The report's major recommendations specifically relevant to quality assurance and genetic testing were that:

• An assessment be made for the applicability of the NHC's New Health Intervention Assessment (NHIA) framework to the assessment of genetic technologies and genetic tests;

• Clinical validity and utility should direct the funding of new genetic tests;

• The peer review process for genetic testing laboratory accreditation be increased from four to two years to accommodate rapid changes in technology;

• IANZ should accredit laboratories according to professional standards, such as those developed by the Human Genetics Society of Australasia; and

• Protocols should be developed for each test approved for use that cover, among other things, consent protocols, when and how the test should be used and sensitivity to cultural issues (particularly those of the Maori community) [[38], p7].

### Overview of Australian and New Zealand reviews

Not surprisingly, given the similarities in their regulatory structures, the Australian and New Zealand reviews identified similar problems in many areas. Two major areas of similarly were that some laboratories providing clinical testing are not required to be accredited; and that there is no effective system in place for ensuring compliance with accreditation standards. A general theme in the reviews was therefore the need to strengthen compliance mechanisms. Finally, the lack of an oversight body for genetic testing was identified as a problem in both New Zealand and Australia. The ALRC recommended the establishment of such a body, and its report provided a detailed account of how this might be achieved and what functions the proposed HGCA should have. New Zealand could draw on this recommendation in establishing a similar body. Alternatively there may be value in Australia and New Zealand examining the option of developing a joint oversight body to advise on issues specific to genetic testing.

In other areas, the reviews differed in their focus. One example is the ALRC Inquiry view that measures should be put in place to address direct to the public test provision. This concern received relatively little attention in the New Zealand reports, although it may well be a problem in the New Zealand context as well.

## Conclusion

Quality assurance is an important aspect of ensuring patient safety and well-being in relation to genetic testing. The reviews conducted of the Australian and New Zealand schemes to promote quality assurance in genetic testing, in general conclude that these schemes are working well. Both countries have taken a proactive stance in recognising the need to continually monitor and evaluate their regulatory frameworks and investigate measures to improve them. Since these reviews have been conducted, governments in both countries have taken steps to address the problems identified. Given the rapid advances in the field of genetic testing, we watch with interest as these governments continue to develop their regulatory systems governing quality assurance of genetic tests to ensure patient safety and wellbeing.

## Competing interests

Imogen Goold was employed as a Legal Officer by the Australian Law Reform Commission from April 2002 to September 2004 and was involved in researching and writing sections of *Essentially Yours: The Protection of Human Genetic Information in Australia*. The remaining author(s) declare that they have no competing interests.

## Authors' contributions

IG carried out reviews of regulations, guidelines and reports, jointly drafted the manuscript and carried out final revisions. AP carried out reviews of regulations, guidelines and reports and jointly drafted the manuscript. SB undertook the initial research and wrote summaries of the research on which the current paper is based, provided feedback on future drafts. AB initiated and oversaw the research, advised on the structure, focus and discussion in the manuscript and provided feedback on drafts. All authors read and approved the final manuscript.
